# State of American Medicine before the Revolution

**Published:** 1842-08

**Authors:** 


### THE WESTERN JOURNAL.

Vol. VI.—No. II.

LOUISVILLE, AUGUST 1, 1842.

### STATE OF AMERICAN MEDICINE BEFORE THE REVOLUTION.

American medicine has few abler advocates or ornaments than the Drs. Beck, to one of whom we are indebted for an excellent address delivered before the Medical Society of New York on the progress of Medicine before the revolution. They have contributed a full share to carry a knowledge of their profession beyond the sea, and to make it to be respected in all countries where it is cultivated as an enlightened and liberal calling. Dr. John B. Beck, in the address before us, has vindicated the claims of American physicians to some improvements in practical medicine heretofore generally conceded to Englishmen. We give one example from among a number:

"Although the physicians in the colonies generally followed the prevalent practice of the mother country, yet they are entitled to the credit of originating some modes of practice of great value. The most important of these is the application of mercury in the treatment of inflammatory complaints. This practice took its origin as far back as the year 1736, and the credit of originality is generally conceded to Dr. Douglass, a physician of Boston, by whom it was used in the angina maligna which prevailed extensively over the colonies at that period, and committed the most dreadful ravages. By Dr. James Ogden, a respectable physician of Long Island, this practice was extensively applied in the same disease about the year 1749.

The preparation of mercury which was used was calomel. In consequence of the success which attended the use of this remedy in this disease, it was shortly after resorted to in other inflammatory complaints; and about the middle of the last century it was in common use, in this country, in pleurisy, pneumonia, rheumatism, and others of the phlegmasiæ. I am aware that the credit of this practice is claimed elsewhere; but there can be no doubt that in its origin it is exclusively American, and that to our colonial physicians the world is indebted for one of the greatest improvements ever made in practical medicine."

The credit of this practice is generally awarded to Dr. Robert Hamilton of Lynn Regis, but Dr. Beck shows that, from Dr. Hamilton's own account, his attention was not called to the practice until the year 1764, and that it had been in very general use in this country many years before.

The only fault of this address is, that it is too short, and this defect the author, we hope, will remedy by future contributions to the history of American Medicine. It is a subject which he seems to be peculiarly qualified by his studies to render interesting and instructive to his young countrymen. He closes his address in the following exulting language which every American will heartily adopt.

"The revolution accomplished, and an independent government established, a new career was commenced. In common with every thing else, medicine felt the sacred impulse, and during the brief period of our independence, how has the scene changed! Instead of the feeble beginnings of one or two institutions, twenty-three well established medical colleges are now to be found in different parts of our country; every city has its hospitals; a thriving professional literature has sprung up among us, and we can now boast of authors whom we are not ashamed to mention along with those of European birth. What nation ever accomplished so much in an equal space of time, and under equal circumstances!"

